# Detection of hepatitis B virus genotypes in a group of hepatitis B virus-infected patients in central and northern Sri Lanka

**DOI:** 10.1099/acmi.0.000838.v3

**Published:** 2024-10-03

**Authors:** T. T. Pattiyakumbura, K. G. K. Malkanthi, W. K. H. Dheerasekara, A. Manamperi, M. A. R. V. Muthugala

**Affiliations:** 1 National Hospital, Kandy 20000, Sri Lanka; 2 Faculty of Allied Health Sciences, University of Peradeniya, Peradeniya 20400, Sri Lanka; 3 Faculty of Medicine, University of Kelaniya, Kelaniya 11300, Sri Lanka

**Keywords:** genotypes, HBeAg, hepatitis B, Sri Lanka, viral load

## Abstract

**Introduction.** Hepatitis B infection causes a spectrum of clinical diseases varying from asymptomatic infection to severe or fulminant acute hepatitis, chronic liver disease, cirrhosis and hepatocellular carcinoma. Hepatitis B virus (HBV) genotypes appear to influence transmission dynamics, clinical outcomes and responses to antiviral therapy. However, hepatitis B genotyping has been poorly investigated in Sri Lanka. This study intended to determine hepatitis B genotypes in a group of HBV-infected people in central and northern Sri Lanka.

**Methodology.** The study was a laboratory-based descriptive cross-sectional study. Initial detection of HBV DNA in 100 EDTA blood samples was done by using a commercially validated quantitative real-time PCR kit. Hepatitis B genotyping was performed by in-house conventional semi-nested multiplex PCR using genotype-specific primers (for genotypes A–F). The serological profile was determined using a commercially validated ELISA/chemiluminescence immunoassay. The results were evaluated for genotype prevalence, viral load association and hepatitis B e antigen (HBeAg) expression in the study population.

**Results and conclusion.** The study detected that genotype C (*n*=38) is most prevalent and infections with multiple genotypes (*n*=52, 52%) were commoner than mono-genotype (*n*=23, 23%) infections. In total, 25% of patients had no detectable genotype among genotypes A–F. The mean viral load in asymptomatic patients with a single genotype was 3.28 log_10_ copies ml^–1^ and in multiple genotypes was 4.18 log_10_ copies ml^–1^ before treatment. Statistical significance was not detected in mean viral loads and HBeAg expression in these two groups. In the future, chronic HBV infection may be effectively treated and managed according to the infected genotype.

## Data Summary

The data generated throughout this study, along with the supporting data, form the basis for the conclusions presented. These data were essential for replicating the described procedures and validating the findings. Detailed methodologies, results and supplementary data have been provided to ensure that the research is transparent and reproducible. These data will enable other researchers to accurately follow the protocol and verify the outcomes described in this article.

## Introduction

Hepatitis B virus (HBV) can cause life-threatening hepatitis, making it a major global health problem. Hepatitis B infection causes a spectrum of clinical diseases varying from asymptomatic infection to severe or fulminant acute hepatitis, chronic liver disease, cirrhosis and hepatocellular carcinoma (HCC) [1]. The virus is mainly transmitted by percutaneous or mucosal contact with contaminated blood or body fluids, or by vertical transmission from an infected mother to a child. The age at which the infection is acquired primarily determines the probability of progression from acute to chronic hepatitis B, with childhood infections carrying a higher risk [2]. The highest prevalence of hepatitis B infection is seen in the WHO Western Pacific and WHO African regions [1]. Although community prevalence of hepatitis B infection in Sri Lanka is modest, 2% according to serology markers, the infection nevertheless poses a considerable risk to those who have underlying comorbidities such as haemodialysis, kidney transplants or multiple transfusions [3]. Hepatitis B immunization campaigns, promoting safe sexual practices and safe injection usage among intravenous drug users, strengthening infection prevention and control practices and injection safety in healthcare institutions, and ensuring safe blood products through pre-transfusion screening may all have contributed to the low prevalence in Sri Lanka. However, Sri Lanka is at risk as it has a population that is closely linked to several moderate- to high-seroprevalence countries, including India (2–4%), China (3%), Singapore (6%) and South Korea (7–8%) [4,8].

The hepatitis B genome can be classified into ten genotypes, A–J, by comparing their nucleotide sequences. There is a significant link between genotype and geographical distribution [9]. Hepatitis B genotypes appear to influence transmission dynamics, clinical outcomes and responses to antiviral therapy [10,11]. A study from more than a decade ago revealed genotypes B, C and D were the predominant genotypes in Sri Lanka. The authors investigated 25 patients with chronic hepatitis B infection whose genotypes were identified between 2007 and 2009. Genotypes G and H were not determined in that study, and genotypes I and J had yet to be discovered at the time of the study, so those two genotypes were also disregarded [12]. Another study identified genotype A as the predominant genotype in two of six surgical patients in the central province of Sri Lanka during the 2007–2011 period [13]. This research was conducted to determine hepatitis B genotypes (A–F) in a group of hepatitis B-infected individuals in central and northern Sri Lanka reported during 2018–2022. Additional objectives were to describe viral load and serological profile in different hepatitis B genotypes in the study population.

## Methodology

**Fig. 1.**
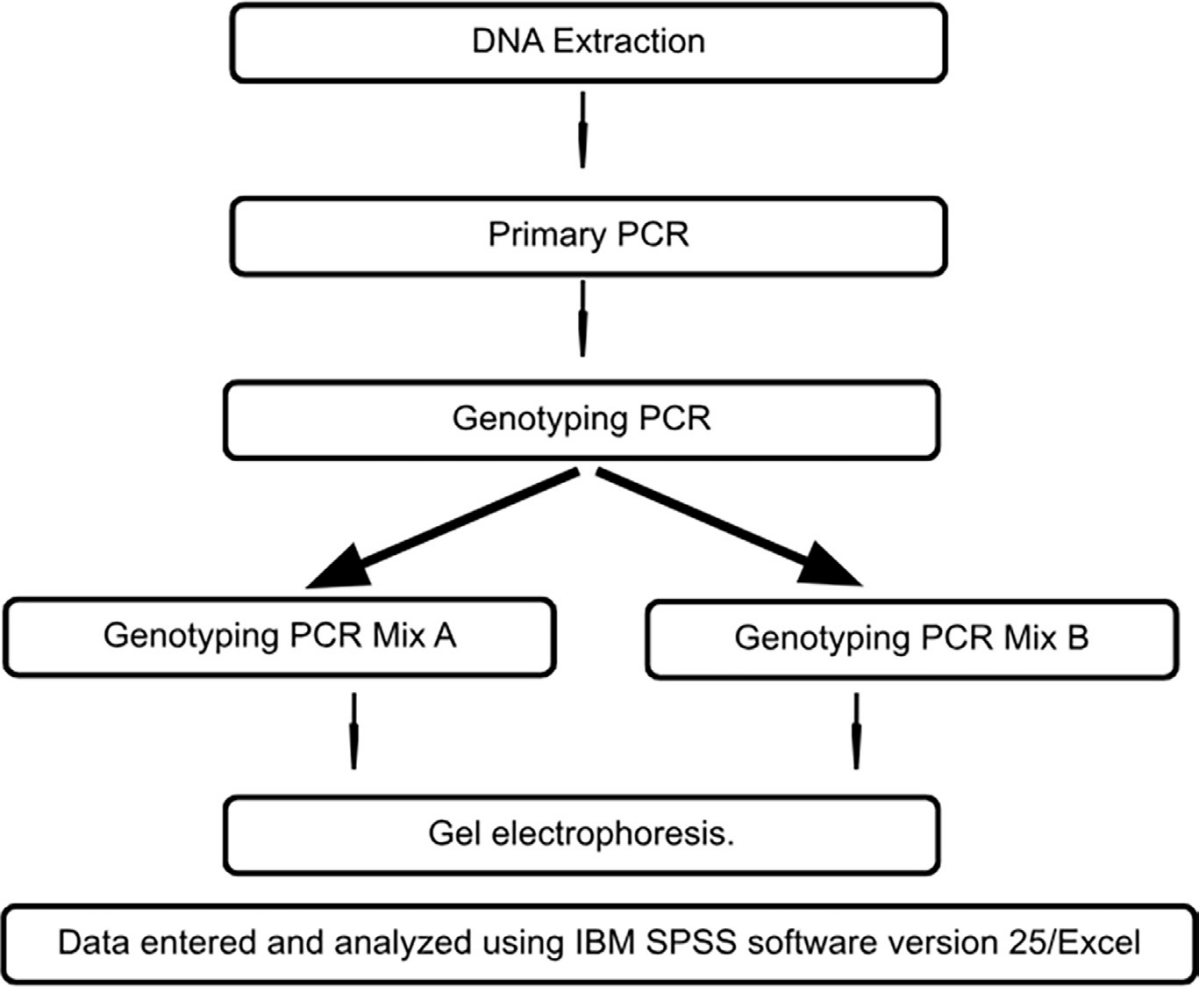
Summary of the laboratory procedure.

This was a laboratory-based descriptive cross-sectional study, conducted at the Regional Virology Laboratory, National Hospital, Kandy, and Molecular Medicine Unit, Faculty of Medicine, University of Kelaniya, Sri Lanka. The consecutive sampling technique was used until a minimum sample size of 100 was achieved from hepatitis B DNA-positive blood samples (RealStar HBV PCR Kit 2.0; Altona Diagnostics) received at Regional Virology Laboratory Kandy, which were referred from hospitals and blood banks in central, northwestern, eastern and northern parts of the country to determine hepatitis B viral loads in known hepatitis B patients. Inadequacy of the sample volume for PCR, and repeated received samples of the same patient were excluded (Fig. 1 provides a summary of the laboratory procedure).

Hepatitis B DNA was extracted from 200 µl of preserved EDTA plasma by using a commercially validated DNA extraction kit according to the manufacturer’s instructions (SpinStar kit 1.0; ADT Biotech). To exclude any contamination, molecular-grade water was extracted along with the samples. Extracted DNA at 60 µl was obtained from each specimen. The first PCR was performed in a 25 µl reaction mixture using 0.25 µM of S1 and P1 outer primers on the Bio-Rad CFX96 PCR Machine (BioRad Laboratories). Each first PCR product was subjected to two second-round PCRs. A 1 µl aliquot of the first PCR product diluted 1:10 was taken for the second PCR. The second PCR had two steps. The first step was conducted using the common universal sense primer (B2) and genotype-specific primers for genotypes A, B and C in ‘Mix A’ (Mix A contained primers B2, BA1R, BB1R and BC1R). The second step was performed using the common universal anti-sense primer B2R and genotype-specific primers for genotypes D, E and F in ‘Mix-B’ (Mix B contained primers B2R, BD1, BE1 and BF1). The following thermal cycles were performed to amplify the first PCR products: denaturation at 94 °C for 20 s, annealing at 58 °C for 20 s and amplification at 72 °C for 30s. All three steps were repeated for 40 cycles. Agarose gel electrophoresis containing ethidium bromide-stained 3% agarose gel was used to separate all PCR products. The genotype of each sample was determined by comparing the migration pattern of a 100 bp marker to genotype-specific bands in each sample in different positions on the gel. A gel imaging system with a UV illuminator (EL4012; Alpha laboratories) was used to visualize the bands. Band sizes for genotypes of mix A as A – 68 bp, B – 281 bp and C – 122 bp, and mix B genotypes as D – 119 bp, E – 167 bp and F – 97 bp were expected (Table 1 gives details of the primer sequences used for genotyping).

**Table 1.** Primer sequences used for genotyping

Primer	Sequence (position, specificity, polarity)
**First PCR**	
P1	5′-TCACCATATTCTTGGGAACAAGA-3′ (nt 2823–2845, universal, sense)
S1–2	5′-CGAACCACTGAACAAATGGC-3′ (nt 685–704, universal, antisense)
**Nested PCR – Mix A**	
B2	5′-GGCTCMAGTTCMGGAACAGT-3′ (nt 67-86, types A to E specific, sense)
BA1R	5′-CTCGCGGAGATTGACGAGATGT-3′ (nt 113-134, type A specific, antisense)
BB1R	5′-CAGGTTGGTGAGTGACTGGAGA-3′ (nt 324–345, type B specific, antisense)
BC1R	5’- GGTCCTAGGAATCCTGATGTTG-3′ (nt 165-186, type C specific, antisense)
**Nested PCR – Mix B**	
BD1	5′-GCCAACAAGGTAGGAGCT-3’ (nt 2979–2996, type D specific, sense)
BE1	5′-CACCAGAAATCCAGATTGGGACCA- 3′ (nt 2955–2978, type E specific, sense)
BF1	5′-GYTACGGTCCAGGGTTACCA-3′ (nt 3032–3051, type F specific, sense)
B2R	5′-GGAGGCGGATYTGCTGGCAA-3′ (nt 3078–3097, types D to F specific, antisense)

Parallel to the patients’ samples, known positive samples for each genotype (A–F) and samples containing molecular-grade water (no template control) were analysed. Following agarose gel electrophoresis of PCR products, the validity of the PCR run was determined by obtaining no band for no-template control samples and genotype-specific bands for known positive samples for each genotype.

Demographic data of the corresponding patient were extracted through the laboratory workbook and from the specimen accompanying request forms. Every day, the database was extensively verified for unintentional errors and plausibility. The IBM Statistical Package for the Social Sciences (SPSS) software version 25 was used to analyse the data. Single and multiple genotype proportion, the association of mean viral load (AltoStar HBV PCR Kit 1.5) and hepatitis B e antigen (HBeAg) expression (LIAISON XL, Diasorin) were determined in single vs multiple genotype groups in asymptomatic patients. These analyses were performed using an independent Student’s *t*-test for mean viral load and Fisher’s exact test for HBeAg expression.

## Results

Results were analysed under several sub-categories: socio-demographic characteristics, patients’ symptomatology, each genotype proportion, and prevalence of single and multiple genotype infection. Asymptomatic patients in the study group were divided into two groups as those with monogenotypic infection and multiple genotypic infection. These two groups were further analysed for HBeAg expression and mean viral load associations.

### Characteristics of the socio-demographic data

**Fig. 2.**
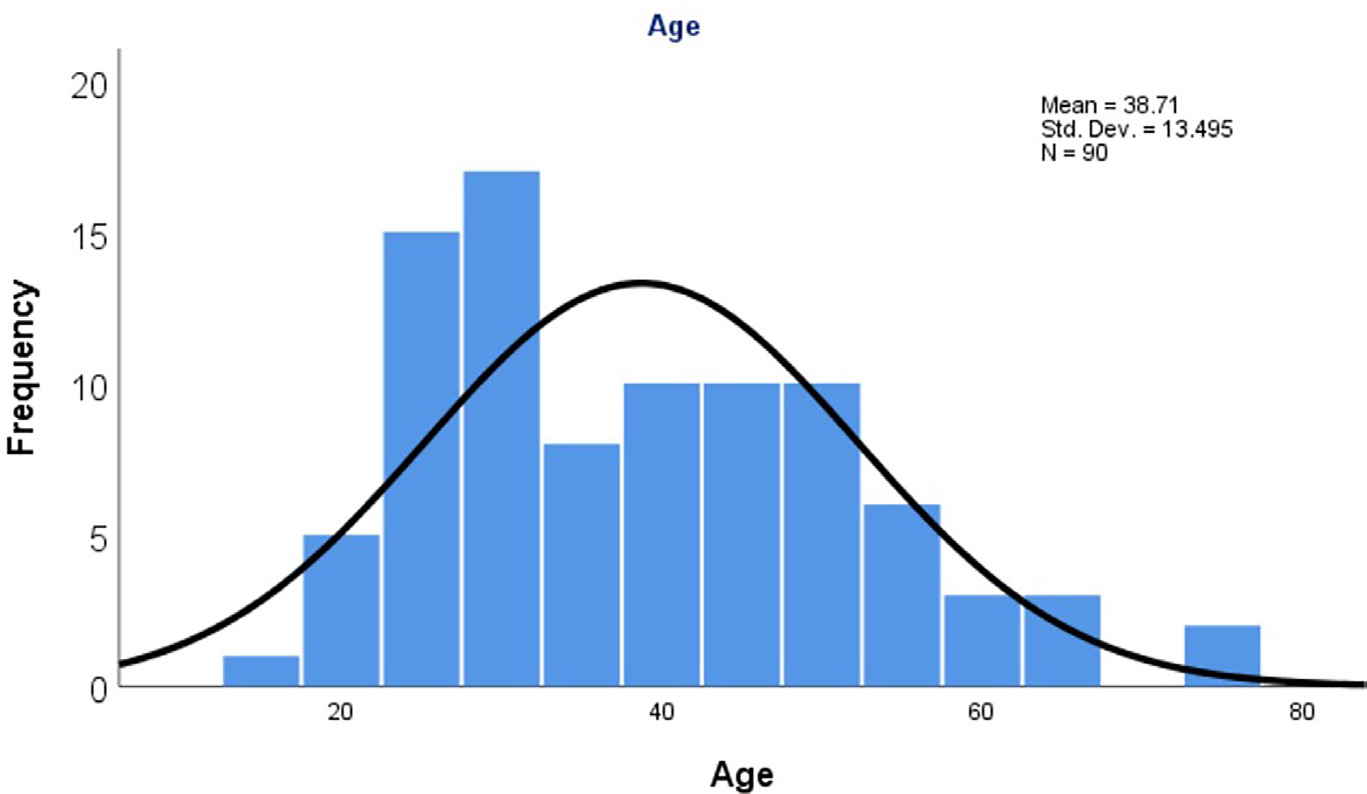
Age distribution of the study population.

Of the 100 specimens, data were not available for 11 specimens. In the available data, the majority of specimens were referred from gastroenterology clinics (55.1%), followed by blood banks (27%), wards (9%), antenatal clinics (3.4%) and outpatient department (2.2%). Coronary care units, sexually transmitted disease clinics and renal units each sent 1.1% of specimens. The 100 samples tested in this study belonged to 100 patients. Among them, ten specimens had data missing for age and gender. In total, 58 (64.4%) were males and 32 (35.6%) were females. The mean (±sd) age was 38.7± 13.4 years with a range of 15–76 years (Fig. 2 illustrates the age distribution of the study population).

### Characteristics of the patients’ symptomatology

Of the 100 patients, only 90 had details of symptomatology. Among them, 49 patients (54. 4%) were symptomatic for hepatitis B infection and 41 patients (45.6 %) were asymptomatic. The remaining patients (10) had no documented details on symptomatology (Table 2 illustrates the proportion of asymptomatic and symptomatic patients).

**Table 2.** Proportion of asymptomatic and symptomatic patients

	Frequency	Percentage
Asymptomatic	41	45.6
Symptomatic	49	54.4
Total	90	100

### Characteristics of HBeAg expression in the population

Only 71 samples had data on serum HBeAg expression. Only 18 (25.4%) of the specimens were positive for HBeAg, while the remaining 53 (74.6%) were negative (Table 3 illustrates HBeAg expression in serum).

**Table 3.** HBeAg expression in serum

	Frequency	Valid percentage
HBeAg negative	53	74.6
HBeAg positive	18	25.4
Total	71	100

### Detection of hepatitis B genotype

**Fig. 3.**
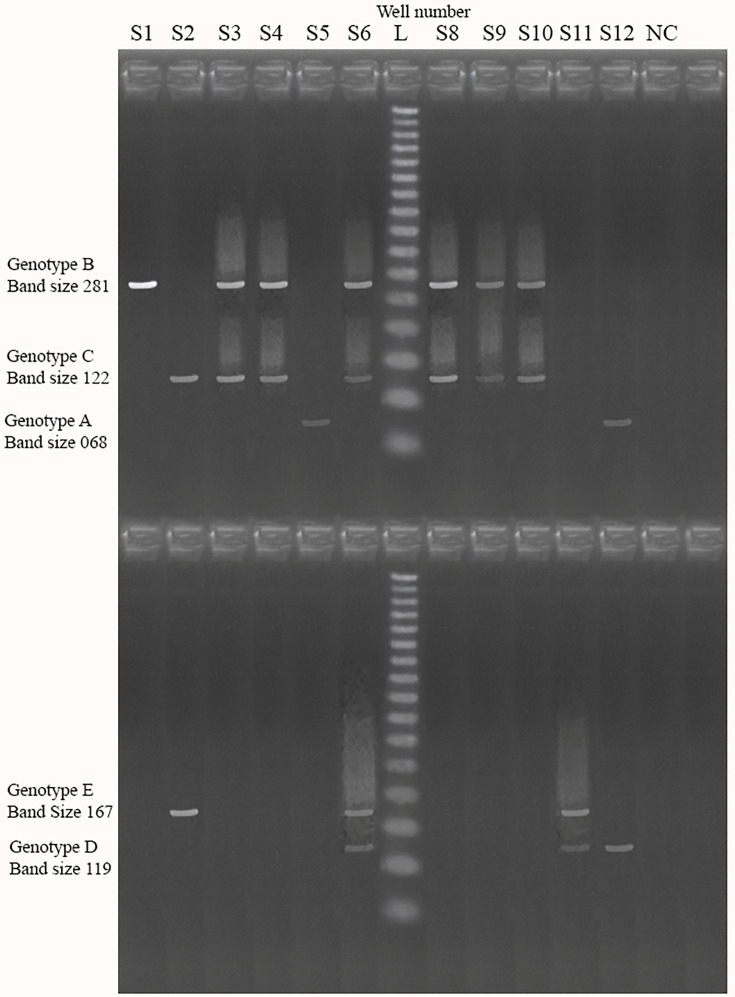
Visualization of genotype-specific bands.

Of 100 HBV-DNA positive blood samples, only 75 were successfully genotyped with genotype-specific primers for genotypes A–F. Among them, genotype C (n=38) was the most prevalent, followed by genotype A (n=29), genotype D (n=29), genotype B (n=21), genotype E (n=20) and genotype F (n=18). Among the 100 specimens, 52 (52%) had multiple genotypes. In contrast, 23 specimens (23%) only had a single genotype (Fig. 3 provides a visualization of genotype-specific bands with gel electrophoresis, Table 4 illustrates the proportion of each genotype and Table 5 illustrates single and multiple genotype combinations).

**Table 4.** Proportions of each genotype

Genotype	No.
A	29
B	21
C	38
D	29
E	20
F	18

**Table 5.** Single and multiple genotypes

No. of genotypes	Frequency	Percentage
Not-typed	25	25
1	23	23
2	26	26
3 or more	26	26
Total	100	100

### Viral load association in single and multiple genotype groups in asymptomatic patients

Comparison of the mean viral load in the single genotype group versus that in the multiple genotype group was performed in asymptomatic patients who had not received antiviral therapy. Specimens with a single genotype (9) had a mean viral load of 3.28 log_10_ copies ml^–1^. Specimens with multiple genotypes (20) had a mean viral load of 4.18 log_10_ copies ml^–1^. An independent Student’s *t*-test was used to compare the mean viral load of these two groups, with statistical significance taken as *P*<0.05. The *P*-value for this comparison was 0.303, df=27 (95% CI). The mean viral load in the single genotype group was not statistically significant from that in the multiple genotype group in asymptomatic patients (Table 6 illustrates the viral load association in single and multiple genotype groups).

**Table 6.** Viral load association in single and multiple genotype groups in asymptomatic patients

Asymptomatic	No.	Mean viral load(log_10_ copies ml^–1^)	*t*-value	*P*-value
Single genotype group	9	3.28	−1.04	0.303
Multiple genotypes group	20	4.18		

### HBeAg expression in single versus multiple genotypes

Patients with asymptomatic infection and who had no prior antiviral therapy were further analysed for HBeAg expression in the single genotype and multiple genotype groups. In specimens with a single genotype (7), the expression of HBeAg was detected in only two specimens (28.6%). In specimens with multiple genotypes (17), HBeAg expression was detected in four specimens (23.5%). Fisher’s exact test was used to analyse the relationship between these two groups of categorical variables, with statistical significance taken as *P*<0.05. The *P* value for this comparison was 0.35. Thus, HBeAg expression in the single genotype group was not statistically significant from that in the multiple genotype group (Table 7 illustrates HBeAg expression in the single versus multiple genotype groups in asymptomatic patients).

**Table 7.** HBeAg expression in single versus multiple genotype groups in asymptomatic patients

Asymptomatic	HBeAg positive frequency	HBeAg negative frequency	Fisher’s exact test (*P*-value)
Single genotype group	2	5	0.35
Multiple genotype group	4	13	

## Discussion

The study found that genotype C (*n*=38) was the most prevalent genotype, followed by genotype A (*n*=29), genotype D (*n*=29), genotype B (*n*=21), genotype E (*n*=20) and genotype F (*n*=18). These results are consistent with the first study describing hepatitis B genotypes in Sri Lanka in the years 2007–2008. In that study, researchers analysed 25 specimens in a group of patients with chronic hepatitis B infection and identified genotypes B–D as the predominant genotypes [12]. According to the WHO Factsheets, the results of this study are consistent with previous findings in countries close to Sri Lanka. The predominant hepatitis B genotypes in India were genotypes D (78%) and A (22%) [14]. In Bangladesh, the predominant genotypes were genotypes C (45.3%) and D (35.8%) [15,17]. However, in Pakistan, the prevalence of genotypes B–F as a whole was only 1.5%, which was inconsistent with the results of our study, where genotype C was identified as the most prevalent genotype [18]. Genotype C (26%) is the most frequent genotype globally and genotypes F–I are the least frequent (<2 %) [19]. This is also consistent with the findings of the present study. Also, genotypes B (30.9%) and C (17.8%) are prevalent in Southeast Asia, China and Japan, which is again consistent with the results of this study [16,20]. Hepatitis B genotypes appear to influence transmission dynamics, clinical outcomes and responses to antiviral therapy. Genotype A has been associated with higher rates of HBeAg and HBsAg loss when treated with IFN compared to genotypes B and D [11]. Studies have shown that hepatitis B genotype B causes a slower rate of cirrhosis, and slower HCC progression compared to genotype C, and that HBeAg seroconversion usually occurs more than 20 years later in people with genotype C than in people with genotypes A, B, D and F. Also, a significant increase in HCC has been reported with genotype C [21]. This study revealed that the predominant genotype is genotype C in the study population, and particular attention should be paid to the management of patients with chronic hepatitis B infection [22].

In this study, hepatitis B infection with a single genotype was found in 23% and infection with more than one (multiple) genotype was found in 52%. A previous study in Sri Lanka also found a high proportion of mixed genotypic infections (B+C, A+D and B+D) in their study population [12]. This result is consistent with previous global studies, which suggest that genotype co-infection is common in chronic HBV infection [23]. Studies have also shown that co-genotypes B and C in Asian-Pacific regions, and co-genotypes A and D in Western countries are common [24]. However, the mean viral load in the single genotype group was not statistically significant from that in the multiple genotype group in asymptomatic patients in our study. According to previous studies, patients with mixed genotypes who were infected with human immunodeficiency virus (HIV) had considerably higher hepatitis B viral load than patients with a single genotype [25]. This result may have been influenced by HIV co-infection and therefore cannot be compared with our study. Also, HBeAg expression in asymptomatic patients with the single genotype group was not significantly different from that in the multiple genotype group. Since prior antiviral therapy in symptomatic patients may affect viral load, statistical analysis was not performed in this group [26].

This study had several strengths and limitations. The main strength was that the samples selected for the study were received from multiple hospitals and clinics across northern and central regions of Sri Lanka, including the Central, North Western, Northern, North Central, Uva and Eastern provinces. It represented six provinces out of a total of nine in Sri Lanka. However, the sample size was one of the major limitations and being a low-seroprevalence country, finding HBV-DNA-positive samples was a challenge during the study period. The study detected only six of the ten genotypes found to date (genotypes A–F). Genotypes G–J have not been determined due to the unavailability of genotype-specific primers. Some specimens did not provide a band on gel electrophoresis. Those samples have been reconfirmed as hepatitis B DNA-positive samples using hepatitis B-specific outer primers (S1, P1) through gel electrophoresis. Reduced sample quality due to inappropriate storage conditions and repeated freeze–thawing may be possible reasons for the lack of a band on gel electrophoresis. The relationship between genotype and clinical outcome, such as the influence of genotype on the development of chronic hepatitis B, the development of HCC and the response to antiviral therapy, was not investigated in this study. As this study was conducted as a retrospective laboratory-based study, patient follow-up during the study period was not feasible.

In conclusion, this is the first study describing each genotype proportion in hepatitis B-infected patients in central and northern Sri Lanka. Also, this study is the first to show an association between the HBV load and HBeAg in single versus mixed genotypes in hepatitis B-infected patients in Sri Lanka. Hepatitis B genotyping was done by in-house conventional semi-nested PCR using genotype-specific primers according to the protocol of Manamperi *et al.* focusing on six major genotypes (A–F) [12]. This technique has higher sensitivity for detecting mixed genotypes and is simple and cost-effective with a high accuracy rate for large population studies. The study found that genotype C is the most prevalent out of the six genotypes tested in the study population. Infections with multiple genotypes were commoner than mono-genotype infections. Also, there was no significant difference in mean viral load and HBeAg expression in asymptomatic patients infected with multiple genotypes and patients infected with the single genotype. In multiple samples, gel electrophoresis revealed non-specific bands. These could represent additional genotypes that were not studied in this study (G–J) or new genotypes that have not yet been identified. The gene sequencing technique may be useful in identifying those genotypes that have yet to be recognized. Also, hepatitis B genotype co-infection commonly found in this study and other studies may play a key role in the origin of new viral strains. Future studies are expected to evaluate the impact of genotype co-infections, the influence of genotype on the development of chronic hepatitis B infection, the development of HCC and the response to antiviral therapy. In the future, chronic hepatitis B infection may be effectively treated and managed according to the infected genotype. Future research on hepatitis B genotype distribution, clinical outcome and response to antiviral therapy in the country as a whole is suggested.

## supplementary material

10.1099/acmi.0.000838.v3 Uncited Table S1.

## Abbreviations

HBeAg: hepatitis B e antigen
HBV: hepatitis B virus
HCC: hepatocellular carcinoma
