# Supplementary material for: Detection of hepatitis B virus genotypes in a group of hepatitis B virus-infected patients in central and northern Sri Lanka
Source: Access Microbiol. 2024 Oct 3;6(10):000838.v3. doi: 10.1099/acmi.0.000838.v3 (PMC11449138; doi:10.1099/acmi.0.000838.v3)
Supplement: Uncited Table S1. [file acmi-6-00838-s001.pdf]

Table-8 Single and multiple genotype combinations in the study population

Genotype combination	Frequency	Percentage %
A	8	10.7
A, B, C	1	1.3
A, B, C, E	1	1.3
A, B, C, D, E, F	2	2.7
A, B, C, D, F	1	1.3
A, B, C, F	1	1.3
A, C	3	4.0
A, C, D	1	1.3
A, C, D, E	2	2.7
A, C, D, E, F	1	1.3
A, C, D, F	5	6.7
A, D	4	5.3
A, D, E	1	1.3
A, D, F	1	1.3
A, F	1	1.3
B	4	5.3
B, C	8	10.7
B, C, D, E	4	5.3
B, C, E	1	1.3
C	5	6.7
C, D, E	1	1.3
C, D, F	1	1.3
C, E	4	5.3
C, F	1	1.3
C, E	1	1.3

D	3	4.0
D, E	1	1.3
D, E, F	2	2.7
D, F	3	4.0
E	1	1.3
F	2	2.7
Total	75	100
